# BMP4/Smad1 Signalling Promotes Spinal Dorsal Column Axon Regeneration and Functional Recovery After Injury

**DOI:** 10.1007/s12035-019-1555-9

**Published:** 2019-03-28

**Authors:** Fatima Farrukh, Elise Davies, Martin Berry, Ann Logan, Zubair Ahmed

**Affiliations:** Neuroscience and Ophthalmology, Institute of Inflammation and Ageing, College of Medical and Dental Sciences, University of Birmingham, Birmingham, B15 2TT UK

**Keywords:** Spinal cord injury, BMP4, Smad1, Dorsal root ganglion neurons, Dorsal column, Axon regeneration

## Abstract

Signalling through the BMP4/Smad1 pathway promotes corticospinal tract axon regeneration and functional recovery in mice. However, unlike humans and rats, mice do not cavitate. Here, we investigated if activation of the BMP4/Smad1 pathway promotes axon regeneration and functional recovery in a rat model that cavitates. We show that dorsal root ganglion neurons (DRGN) in injury models, including the non-regenerating dorsal column (DC) and the regenerating sciatic nerve (SN) crush and preconditioning (p) SN + DC (pSN + DC) paradigms, regulate the BMP4/Smad1 signalling pathway. For example, mRNA expression of positive regulators of the BMP4/Smad1 pathway was highly up-regulated whilst negative regulators were significantly down-regulated in DRGN in the regenerating SN and pSN + DC models compared to non-regenerating DC models, matched by concomitant changes in protein expression detected in DRGN by immunohistochemistry. BMP4 peptide promoted significant DRGN survival and disinhibited neurite outgrowth in vitro, whilst AAV-BMP4 delivery in vivo stimulated DC axon regeneration and functional recovery in a model that cavitates. Our results show that activation of the BMP4/Smad1 pathway is a potential therapeutic target in the search for axon regenerative signalling pathways in the CNS.

## Introduction

Several signalling pathways have been associated with axon regeneration after spinal cord injury (SCI). These include the phophoinositide-3-kinase (PI3K) and extracellular receptor kinase (ERK) pathways that are essential for axon assembly and neurotrophin-induced axonal branching [1–3]. The PI3K-Akt pathway also regulates local protein synthesis via the mammalian target of rapamycin (mTOR) signalling pathway with adult neurons requiring mTOR signalling to promote axon regeneration [4]. It is not known if mTOR signalling plays a role in the regeneration of ascending long-tract dorsal root ganglion neuron (DRGN) dorsal column (DC) projections, but an mTOR independent pathway is implicated, since pSN also promotes DC axon regeneration [5, 6]. The genes of many PI3K independent axogenic signalling proteins are transcribed in axotomised DRGN [7–9]. Prominent among the latter is the bone morphogenetic protein 4 (BMP4)/mothers against decapentaplegic homologue 1 (Smad1) pathway. Smad1 activation, nuclear accumulation and gene transcription require BMP receptor (BMPR) binding and histone 4 (H4) acetylation [10] when BMP/Smad and PI3K/Akt pathways interact to effect axogenesis. For example, (1) PI3K/Akt induces nuclear localisation of BMP4-activated Smad1 and regulates Smad induction of axon regeneration-related transcription factors and (2) BMP4 activates Akt by suppression of PTEN, stimulation of MAPK and autocrine induction of growth factor secretion [11, 12].

BMP4/Smad1 signalling stimulates peripheral DRGN axon growth developmentally, and down-regulation of the pathway with age correlates with a decline in axon growth potential [13]. SN axotomy up-regulates Smad1 [10] and BMP2/4 injected into DRG potentiates SN growth [14]. DRGN neurite outgrowth is suppressed after knockdown of the BMP co-receptor (repulsive guidance molecule b-RGMb), and conversely, inhibition of the BMPR antagonist Noggin in mature DRGN in vivo potentiates SN axon regeneration [15]. The inactivation of Smad1 in DRGN after DC lesions correlates with failed axon regeneration, whereas DRG injection of BMP4 activates Smad1 and promotes DC axon regeneration in the lesioned adult mouse cord [13].

However, the mouse cord does not cavitate after SCI whereas the rat cord does [16], and hence, the impact of BMP4/Smad1 activation in a model that cavitates as in humans remains to be investigated. Here, we used the rat model, which cavitates, to determine the contribution of the BMP4/Smad1 signalling pathway on DC axon regeneration and return of locomotor and sensory function. We show that BMP4/Smad1 is activated when DRGN axons regenerate after an SN and pSN + DC lesions, BMP4 peptide promotes disinhibited DRGN neurite outgrowth in the presence of inhibitory CNS myelin extracts (CME), whilst BMP4 over-expression in DRGN in vivo enhances DC axon regeneration and promotes functional recovery in adult rats, despite the presence of spinal cord cavities. These results suggest that the BMP4/Smad1 pathway is a potential target for therapeutic intervention in SCI patients.

## Methods

### Animals

Adult (6–8-week old) female Sprague-Dawley rats (180–250 g) (Charles River, Margate, UK) were used. Animals were maintained in the animal facility of University of Birmingham, UK, under standard conditions (21 °C, 12-h light-dark cycle) with free access to food and water. All surgery was performed in strict accordance to the guidelines of both the UK Animals Scientific Procedures Act, 1986 and the Revised European Directive 1010/63/EU. Experiments were licenced by the UK Home Office and ethically approved by the University of Birmingham’s animal welfare and ethical review board. Experiments also conformed to the recommendations of the use of animals by the Federation of the European Laboratory Animal Science Associations.

### Experimental Design

**Fig. 1:**
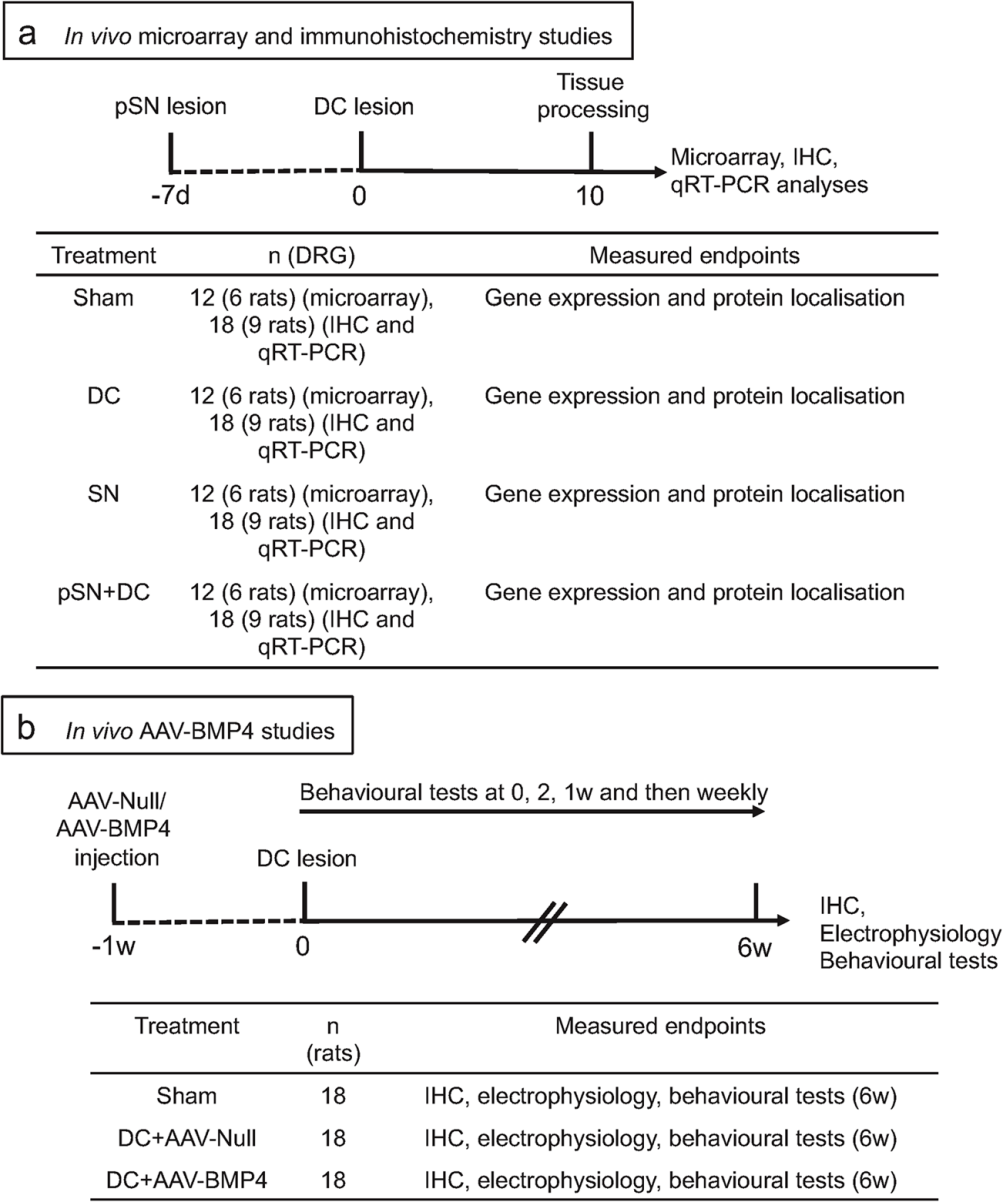
Experimental design of **a** in vivo microarray and immunohistochemistry studies and **b** the in vivo AAV-BMP4 study

For in vivo microarray experiments, four adult female Sprague-Dawley rats/group (*n* = 12 rats/group (3 independent repeats)) were randomly assigned to (1) uninjured intact controls (IC), (2) sham-treated controls (sham: partial laminectomy but no DC crush injury), (3) non-regenerating DC model (DC) that received a DC lesion alone, (4) regenerating SN model (SN) that received a SN lesion alone, and (5) regenerating pre-conditioning SN lesion (pSN) + DC injury model (pSN + DC-pSN lesion performed 7 days before the DC lesion). Tissues were harvested at 10 days after DC, SN and pSN + DC lesions for the microarray, qRT-PCR and immunohistochemistry studies (Fig. 1a) and at 6 weeks after DC lesion for the in vivo adeno-associated virus 8 (AAV8; AAV serotype 8) delivered BMP4 (AAV-BMP4) studies (Fig. 1b). AAV8 has previously been shown by others and ourselves to preferentially transduce large-diameter neurons that form the ascending tracts of the DC [17]. All in vivo experiments, except the microarray study, contained six rats/group, repeated on three independent occasions (total *n* = 18 rats/group/test) and performed by an investigator masked to the treatment conditions. Ten days after DC lesion was chosen since Q-PCR and immunohistochemistry studies at 1, 3, 5, 7, 10 and 17 days after DC lesion showed the greatest gene/protein changes between groups (not shown).

To test the potential of AAV-BMP4 to disinhibit DC axon regeneration and enhance functional recovery in vivo, groups of six adult female Sprague-Dawley were randomly assigned to treatment groups. At 1 week before DC injury (week 1), groups of animals received intra-DRG injection of either control AAV8-Null (Vector Biolabs, Malvern, PA, USA; AAV serotype 8 with a CMV promoter but no transgene) (DC + AAV-Null) or AAV-BMP4 (DC + AAV-BMP4) (Fig. 1b). After 7 days, a group of animals was designated as sham-treated control (sham). Animals from each group received either a sham (partial laminectomy and exposure of the spinal cord but no DC lesion) or DC injury (partial laminectomy followed by crush injury of the DC tracts) [16] 7 days later. Animals were allowed to survive for 6 weeks during which behavioural tests were performed by individuals masked to the treatment conditions at baseline, 2 days and then weekly at 1–6 weeks after injury [18, 19]. Electrophysiology was performed at 6 weeks after injury before harvesting tissues for immunohistochemistry (Fig. 1b) [19]. All in vivo experiments were repeated on three independent occasions (total *n* = 18 rats/group/treatment).

### Surgery and Tissue Harvesting

All animals were injected with Buprenorphine to provide analgesia before anaesthesia using 5% isoflurane with 1.8 l/min O_2_. After a partial laminectomy, DC were crushed bilaterally at the level of T8 using calibrated watchmaker’s forceps [16, 20, 21]. The tips of the forceps, separated to a width of 1 mm, were inserted into the cord through the dorsal meninges to a depth of 1 mm and the DC crushed by tip approximation. The left SN was exposed at mid-thigh level and crushed using fine forceps at the level of the sacrotuberous ligament. In the pSN + DC model, SN were crushed 1 week before DC crush injury. Rats were housed under standard conditions after surgery along with their cage mates in groups of four rats. For the microarray and immunohistochemistry studies, rats were killed at 10 days after DC lesion by CO_2_ exposure when L4/L5 DRG was harvested from the ipsilateral (treated) and contralateral (untreated) sides. DRG were snap frozen in liquid N_2_ before RNA extraction, and for immunohistochemistry, animals were intracardially perfused with 4% formaldehyde in phosphate-buffered saline (PBS) and both ipsilateral and contralateral L4/L5 DRG processed as described below.

### Intra-DRG Injections

The left L4/5 DRG were exposed and injected as described by us previously [17] with either 10^13^ viral genomes of AAV-Null (control; Vector Biolabs) or AAV-BMP4 (gift from Hongyang Zou, Mount Sinai School of Medicine, New York, USA) in a total volume of 5-μl sterile PBS using a glass micropipette.

### Microarray Analysis

RNA was extracted from a total of 12 DRG/treatment (*n* = 4 DRG/treatment, 3 independent repeats) harvested at 10 days after DC lesion from each experimental group detailed above using the RNeasy kit (Qiagen Ltd., Crawley, UK) according to the manufacturer’s instructions. The rat genome AROS™ V3.0 set (Operon Biotechnologies GmbH, Cologne, Germany) containing 29,962 long-mer probes representing 22,012 genes and 27,044 gene transcripts was used for the microarray analysis (Read et al., 2009). Briefly, either Cy3- or Cy5-labelled oligonucleotide probes were hybridised and slides were scanned with an Axon GenePix 4000B scanner (Molecular Devices Ltd., Berkshire, UK); background fluorescence values for Cy3 and Cy5 channels were subtracted and data analysed using GeneSpring GX7 (Agilent, Berkshire, UK) normalised by the Lowess method. Data were filtered below the *P* < 0.05 threshold, and fold changes > 2 above sham control levels were taken as significant. Each condition was replicated × 4 in duplicate.

### Quantitative Real-Time PCR

RNA was extracted from *n* = 6 DRG/treatment, which were pooled together at 10 days after DC lesion, using the RNeasy kit (Qiagen Ltd., Crawley, UK) according to the manufacturer’s instructions. Experiments were repeated on three independent occasions with total *n* = 18 DRG/treatment. Selected genes of the BMP4/Smad1 pathway were validated by Q-PCR using pre-validated primer sequences (Table 1) from complementary DNA prepared from extracted mRNA, and Q-PCR was performed using LightCycler Q-PCR machine (Roche, Burgess Hill, UK) following previously published methods [22]. Fold changes were computed using the ΔΔCt method and the mean ± SEM is presented from the three independent repeats.

**Table 3:** Microarray analysis to show fold-differences in BMP4/Smad1 pathway genes compared to sham controls in rat DRGN 10 days after DC, SN and pSN + DC lesions

Gene	Description	DC	SN	pSN + DC
*smad 1*	Mothers against decapentaplegic homologue 1	1.03 ± 0.05	3.01 ± 0.04***	3.19 ± 0.04***
*smad 2*	Mothers against decapentaplegic homologue 2	1.03 ± 0.05	3.01 ± 0.04***	3.19 ± 0.04***
*smad* 3	Mothers against decapentaplegic homologue 3	1.11 ± 0.10	1.04 ± 0.11	1.06 ± 0.04
*smad 4*	Mothers against decapentaplegic homologue 4	1.09 ± 0.03	2.01 ± 0.01**	2.00 ± 0.02**
*smad* 5	Mothers against decapentaplegic homologue 5	1.01 ± 0.03	3.58 ± 0.02***	3.30 ± 0.03***
*smad 6*	Mothers against decapentaplegic homologue 6	1.01 ± 0.10	1.01 ± 0.05	1.02 ± 0.04
*smad 7*	Mothers against decapentaplegic homologue *7*	1.09 ± 0.06	1.05 ± 0.08	1.03 ± 0.03
*smad* 8	Mothers against decapentaplegic homologue 8	0.99 ± 0.13	2.01 ± 0.02**	2.30 ± 0.03**
*tgfβ1*	Transforming growth factor *β*1	1.09 ± 0.10	1.41 ± 0.06	1.55 ± 0.01
*tgfβ2*	Transforming growth factor *β*2	1.09 ± 0.10	2.41 ± 0.16	2.35 ± 0.08
*bmp-2*	Bone morphogenetic protein 2	1.01 ± 0.02	1.41 ± 0.06	1.55 ± 0.03
*bmp-4*	Bone morphogenetic protein 4	1.09 ± 0.10	6.41 ± 0.16****	6.66 ± 0.11***
*bmp-7*	Bone morphogenetic protein *7*	1.00 ± 0.01	1.09 ± 0.10	1.06 ± 0.02
*smurf1*	Bone morphogenetic protein *7*	1.00 ± 0.01	1.09 ± 0.10	1.06 ± 0.02
*smurf2*	Bone morphogenetic protein *7*	1.04 ± 0.11	1.03 ± 0.09	1.01 ± 0.08
*nogg*	Noggin	1.01 ± 0.08	−2.01 ± 0.09**	− 2.05 ± 0.15**

Means ± SEM are shown from four different samples run in duplicate. **P < 0.001, ***P < 0.0001. Fold difference of > 2 were considered significant

### Immunohistochemistry of DRG

Ipsilateral and contralateral L4/L5 DRG (*n* = 6 DRG/treatment, 3 independent repeats; total *n* = 18 DRG/treatment) from perfusion-fixed rats were cryoprotected through a graded series of sucrose solutions and blocked in optimal cutting temperature (OCT) compound (TAAB Laboratories, Peterborough, UK). DRG cryosections were adhered onto charged glass slides and sections from the middle of each DRG were chosen for immunohistochemistry. After washing in PBS, non-specific antibody binding was blocked with 3% bovine serum albumin diluted in PBS containing 0.1% Triton-X100 before overnight incubation at 4 °C with the relevant primary antibodies (Table 2). Regenerating axons in the DC were detected using GAP-43 immunohistochemistry since Cholera toxin B labelling in our hands did not label regenerating axons by retrograde transport labelling in the rat [23], despite others demonstrating successful labelling [6, 24]. Sections not exposed to primary antibody were included as negative controls in each run, washed in PBS, incubated with relevant secondary antibodies conjugated to either Alexa488 or Alexa594 for 1 h at room temperature (RT; Table 2), washed in several changes of PBS and mounted using Vectashield containing DAPI (Vector Laboratories, Peterborough, UK). Sections were examined using a Zeiss epi-fluorescent microscope attached to an Axiocam HRc run by Axiovision software (all from Zeiss, Hertfordshire, UK). Negative controls were used to set the background threshold level for each antibody before image capture.

**Table 1:** List of primers used for the BMP4/Smad1 pathway

Gene	Primer sequence	Ref
*smad1*	Forward: 5′-ACCTGCTTACCTGCCTCCTG-3′	Kuo et al., 2011
	Reverse: 5′-CATAAGCAACCGCCTGAACA-3′	
*smad5*	Forward: 5′-CTGGGATTACAGGACTTGACC-3′	Kuo et al., 2011
	Reverse: 5′-AAGTTCCAATTAAAAAGGGAGGA-3′	
*smad8*	Forward: 5′-GTATCATCGCCAGGATGTCA-3′	Yew et al., 2005
	Reverse: 5′- TGTGGGGAGCCCATCTGAGT-3′	
*bmp-4*	Forward: 5′ GGCAGAGGAGGAGGGAGGGAGGGAAGGAGC-3′	Kawai and Sugiura, 2001
	Reverse: 5′-CAGTAGCGGGCTCGCCAGCAGCAGCTCCTG-3′	
*creb-p*	Forward: 5′-AAGCTGAAAGTCAACAAATGACAGTT-3′	Shankar et al., 2005
	Reverse: 5′-TGGACTGTCTGCCCATTGG-3′	
*mkk3/6*	Forward: 5′-GGCCCCTGAAAGAATAAACCC-3′	Galan-Moya et al., 2011
	Reverse: 5′-CGAAGGATGGCCAACTCAATC-3′	
*gapdh*	Forward: 5′-ACCACAGTCCATGCCATCAC-3′	Xu et al., 2010
	Reverse: 5′-TCCACCACCCTGTTGCTGTA-3′	

### Primary DRGN Cultures

Primary adult rat (Sprague-Dawley rats, 170–220 g, Charles River) DRGN were prepared from intact DRG according to our previously published methods [25]. DRG cells from ipsilateral and contralateral L4–7 DRG pairs were dissociated using 0.025% collagenase (Sigma, Poole, UK) and plated (500/well) in supplemented neurobasal-A (NBA; containing B-27 supplement and L-glutamine; all from Invitrogen, Paisley, UK), plated in 8-well chamber slides pre-coated with 100 μg/ml poly-D-lysine plus 10 μg/ml of laminin (both from Sigma) and, after settling overnight, incubated with appropriate treatments for 3 days at 37 °C and 5% CO_2_, as described below.

### Treatment of DRGN Cultures with BMP4 Peptide

To establish if BMP4 promoted disinhibited DRGN neurite outgrowth, cultures prepared in 8-well chamber slides as described above were treated with NBA alone (control) or with NBA plus 50, 100, 150 and 200 ng/ml of BMP4 peptide (Peprotech, London, UK), in the presence of 200 μg/ml CME [26]. Cultures were incubated with appropriate treatments for 3 days before fixation in 4% paraformaldehyde and subjected to immunocytochemistry as described below. All slides were masked after treatment for analysis by a second unbiased investigator. Four days later, immunocytochemistry for βIII-tubulin was then used to quantify DRGN neurite outgrowth as described below. Individual treatments in each experimental run were performed in triplicate and runs were repeated on three independent occasions (total *n* = 9 wells/treatment).

### Immunocytochemistry of Cultured DRGN

After 4 days of treatment, DRGN cultures were immersion fixed in 4% formaldehyde for 10 min at RT, permeabilised and non-specific binding blocked using 3% BSA containing 0.1% Triton X-100 in PBS. After incubation with mouse anti-βIII-tubulin antibody (to label DRGN; Table 2) for 1 h at RT, cells were washed in PBS, incubated with Alexa 488 anti-mouse secondary antibody (Table 2) for 1 h at RT, washed in PBS and mounted using Vectashield containing DAPI (Vector Laboratories, Peterborough, UK) and stored at 4 °C until required. Negative staining controls were included in each run in which the primary antibody was omitted and used to set the background fluorescence thresholds for each antibody when capturing images.

### DRGN Survival and Neurite Outgrowth

With the experimenter masked to the treatment conditions, mean numbers of βIII-tubulin^+^ DRGN with neurites and mean neurite lengths were quantified in nine quadrants/well using a Zeiss Axioplan 2 fluorescent microscope equipped with an Axiocam HRc and Axiovision Software (all from Zeiss, Hertfordshire, UK) [25]. The longest neurite was measured using Axiovision from at least 30 DRGN/well/treatment, whilst total DRGN counts to assess survival were made over the entire well for each treatment condition (*n* = 3 wells/treatment, 3 independent repeats; total *n* = 9 wells/treatment).

### BMP4 Enzyme-Linked Immunosorbent Assay

The levels of BMP4 in DRG and in the spinal cord lesion-site tissue were detected by ELISA using a rat BMP4 ELISA kit, according to the manufacturer’s instructions (ElisaGenie, London, UK). DRG (*n* = 6/treatment, 3 independent repeats; total *n* = 18 DRG/treatment) and an area of T8 spinal cord (*n* = 6/treatment, 3 independent repeats; total *n* = 18 cords/treatment) that encompassed the lesion site (1 cm in length × 0.5 mm centred on the lesion epicentre) were collected, weighed and equal amounts were homogenised in 1 ml of tissue extraction buffer (100 mM Tris, pH 7.4, 150 mM NaCl, 1 mM EGTA, 1 mM EDTA, 1% Triton X-100 and 0.5% sodium deoxycholate). Samples were kept at 4 °C for 30 min before clarification by centrifugation at 13,000 rpm at 4 °C for 15 min. ELISA was performed on 10 μl of each sample, in duplicate and repeated on three independent occasions.

### Quantification of DC Axon Regeneration

Numbers of regenerating axons in the DC were quantified as previously described [19, 27]. Composites of serial parasagittal sections through the entire DC were reconstructed by collecting images of GAP-43-labelled axons in the spinal cord from all serial 50-μm-thick sections (∼ 70–80 sections/cord; *n* = 6 rats/treatment, 3 independent repeats; total *n* = 18 rats/group). On each reconstructed composite image, the number of GAP43^+^ axons intersecting a dorsoventral line drawn across the cord was counted at intervals of 2 mm from 4 mm above to 6 mm below the lesion site. Axon number was calculated as % axons seen at 4 mm above the lesion, where the DC was intact. The distance beyond the epicentre of the lesion towards the rostral end was scored as positive and the caudal end as negative distances.

### Electrophysiology

Compound action potentials (CAP) were recorded at 6 weeks after DC + AAV-Null/BMP4 treatment, as previously described [19, 28]. The treatment status of the animal masked from the investigator, sham controls and animals from DC + AAV-Null and DC + AAV-BMP4 groups (*n* = 6 rats/group, 3 independent repeats; total *n* = 18 rats/group) were deeply anaesthetised using 5% isofluorane, and deep anaesthesia was maintained with 1.5% isofluorane for the duration of the experiment, verified by the absence of a withdrawal reflex to a peripheral pinch. Heart rate of approximately 360 beats/min was carefully monitored and body temperature was controlled using a feedback-controlled thermal blanket set at 37 °C. Rats were fixed in a Kopf stereotaxic apparatus (Kopf Instruments, Tujunga, CA, USA), a midline incision was made through the skin, a laminectomy was performed and the dura was cut to expose the thoracic and lumbar spinal cord regions. Spinal cords were bathed in warm mineral oil (37 °C). Silver wire electrodes (0.01″ diameter; A-M Systems, Carlsborg, WA, USA) insulated except at the tip were used to stimulate the DC axons at L1/2 and record CAP at T10-T9 at the surface of the midline spinal cord. Pancuronium bromide (0.3 mg/kg, Sigma) was injected intraperitoneally to minimise muscular contractions throughout the experiment. The signal from the recording electrode was amplified with filters set at 300–3000 Hz, collected and Spike 2 software was used for data analysis (Cambridge Electronic Design, Cambridge, UK). Stimulating single current pulses (0.05 ms) were applied in increasing increments (0.2, 0.3, 0.6, 0.8 and 1.1 mA), recorded at the detecting electrode and the amplified signals were analysed using Spike 2 software (Cambridge Electronic Design). CAP amplitudes and areas were then calculated. At the end of each experiment, the dorsal half of the spinal cord was transected between stimulating and recording electrodes to confirm CAP abolition.

### Functional Testing

Tape removal (sensory) and horizontal ladder crossing (locomotor) functional tests after DC lesions and treatment were carried out as described previously by us and others [18, 19]. Functional deficits using the DC injury model are mild, and hence, we used the horizontal ladder crossing and tape removal tests as two of the five tests described by Fagoe et al. [18] to reliably detect functional deficits after injury. Fagoe et al. [18] and we [19] have shown previously that functional deficits can be reliably detected using these specific tests in the DC injury model. Briefly, animals (*n* = 6 rats/group, 3 independent repeats; total *n* = 18 rats/group) received training to master traversing the rope and ladder for 2 weeks before functional testing. All functional tests were performed 2–3 days before injury to establish baseline parameters. Animals were then tested 2 days after DC lesion and then weekly for 6 weeks at the same time of day and each test performed for three individual trials. For the *horizontal ladder test*, the number of slips for each run was averaged by the total number of steps to calculate the mean error rate. For the *tape removal test*, the time it took for the animals to detect and remove a piece of sticky tape attached to its extended hind paw was recorded and used to calculate the mean sensing time.

### Statistical Analyses

Where indicated, significant differences on means ± SEM were calculated between samples using SPSS Version 22 (IBM, NJ, USA) one-way analysis of variance (ANOVA) with *post hoc* testing using Dunnet’s method.

For the ladder crossing functional test, data was analysed using R package (www.r-project.org) as described previously [18, 19]. Briefly, whole time-course testing of lesioned and sham-treated animals were compared using binomial generalised linear mixed models (GLMM), fitted in R using package *lme4* with the *glmer* function and *P* values calculated using parametric bootstrap. For the tape removal test, linear mixed models (LMM) were calculated by model comparison using the package *pbkrtest*, with the Kenward-Roger method [18, 19]. Individual time-points were also compared using independent sample *t* test without correction.

## Results

### BMP4/Smad1 Pathway Activation in DRGN

**Fig. 2:**
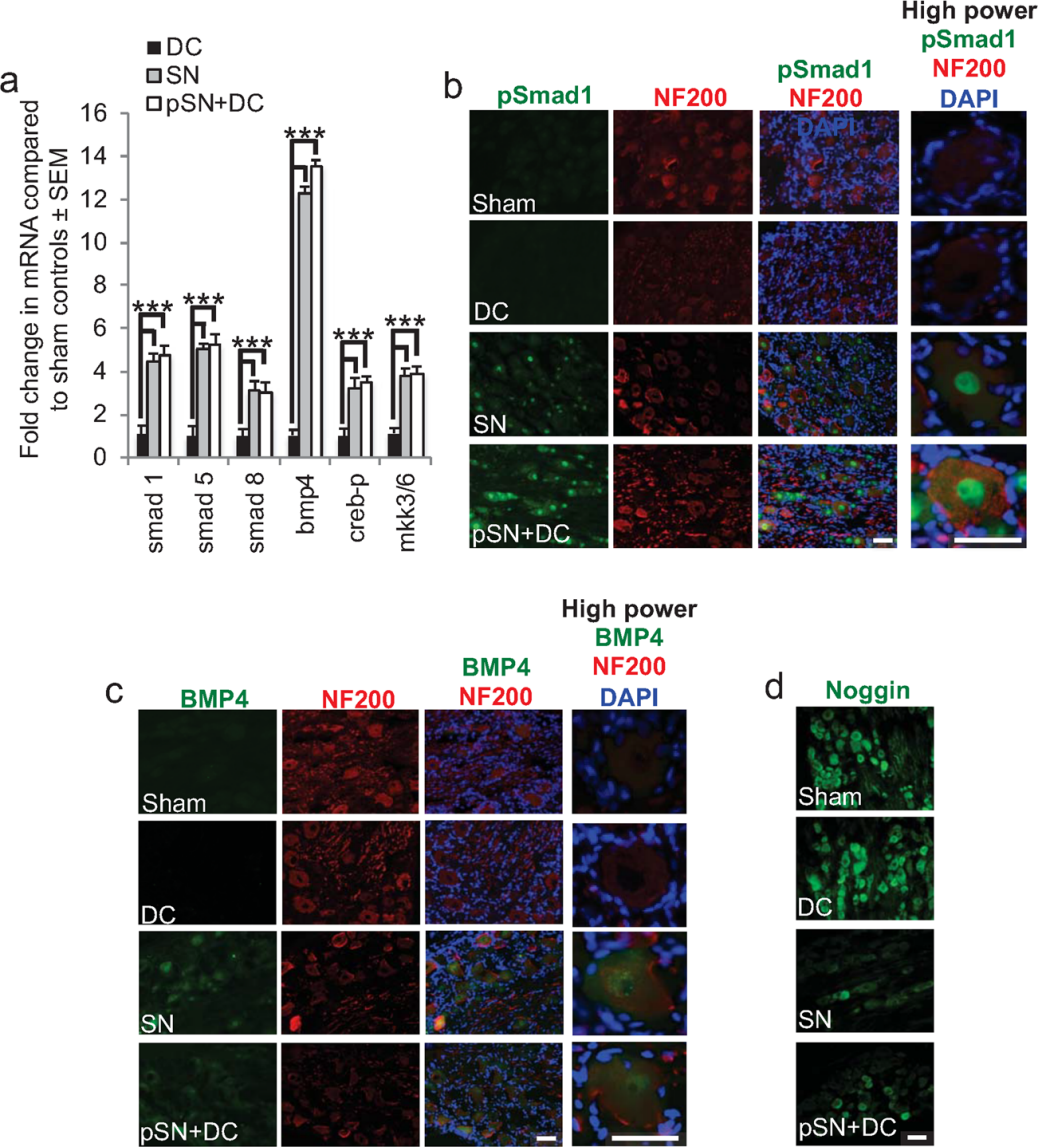
Changes in Smad1/BMP4 signalling pathway. **a** Q-PCR confirmed good correlation between microarray and Q-PCR data. **b** Immunohistochemistry for pSmad1 (green) in sections of regenerating (SN, pSN + DC) and non-regenerating (IC, DC) NF200^+^ DRGN (red) and of satellite cells (DAPI^+^ nuclei—blue). High levels of pSmad1 immunoreactivity were detected in occasional nuclei of regenerating SN and many nuclei of pSN + DC DRGN. **c** Immunohistochemistry for BMP4 (green) in sections of regenerating (SN, pSN + DC) and non-regenerating (IC, DC) NF200^+^ DRGN (red) and of satellite cells (DAPI^+^ nuclei–blue). BMP4 immunoreactivity was detected in most regenerating SN and pSN + DC DRGN. **d** Immunohistochemistry for Noggin in sections of regenerating (SN, pSN + DC) and non-regenerating (IC, DC) DRGN. Noggin immunoreactivity was highest in sham and DC whilst lower levels of immunoreactivity were detected in regenerating SN and pSN + DC DRGN. Scale bars in **b**–**d** = 50 μm

Microarray data showed that the levels of mRNA for *smad1*, *smad2*, *smad4*, *smad5*, *smad8* and *bmp4* were all up-regulated between 2.0- and 6.7-fold compared to sham controls, whilst *noggin* was 2-fold down-regulated, in the DRG from both the SN and pSN + DC models (Table 3), but there was no change in these mRNA levels in DRG from the DC model. Confirmation of the heightened levels of BMP4/Smad1 mRNA in regenerating DRGN was provided by Q-PCR, which reflected changes observed by microarray (Fig. 2a). These results indicated that the BMP4/Smad1 signalling pathway was highly active in DRG after SN and pSN + DC injury when both SN and DC DRGN axonal projections were regenerating.

**Table 2:** List of primary and secondary antibodies used in this study

Antibody	Source	Dilution
Primary antibodies		
Mouse anti-βIII-tubulin	Sigma, Poole, UK	1:400
Rabbit anti-pSmad1	Cell Signalling Technology, Danvers, MA, USA	1:200
Rabbit anti-BMP4	Abcam, Cambridge, UK	1:400
Rabbit anti-Noggin	Abcam, Cambridge, UK	1:400
Mouse anti-NF200	Sigma, Poole, UK	1:400
Mouse anti-GAP-43	Invitrogen, Paisley, UK	1:200
Rabbit anti-GFAP	Sigma, Poole, UK	1:400
Secondary antibodies		
Alexa-488 anti-rabbit	Invitrogen, Paisley, UK	1:400
Alexa-488 anti-mouse	Invitrogen, Paisley, UK	1:400
Alexa-594 anti-mouse	Invitrogen, Paisley, UK	1:400
Alexa-594 anti-rabbit	Invitrogen, Paisley, UK	1:400

### Immunohistochemistry of Smad1, BMP4 and Noggin

In both sham controls and DC models, pSmad1 (Fig. 2b) and BMP4^+^ immunoreactivity (Fig. 2c) were not detected in DRG cells, but in SN and pSN + DC models, both were expressed in DRGN soma and nuclei, the former in greater amounts than the latter, but both proteins were absent from satellite cells. On the other hand, Noggin^+^ immunoreactivity was present in DRGN somata in sham controls and DC, but lower levels were detected in regenerating SN and pSN + DC models (Fig. 2d). These results confirmed the highly activated state of BMP4/Smad1 signalling in DRGN of the regenerating SN and pSN + DC models.

### BMP4 Disinhibited DRGN Neurite Outgrowth In Vitro and DC Axon Growth In Vivo, Independent of mTOR

**Fig. 3:**
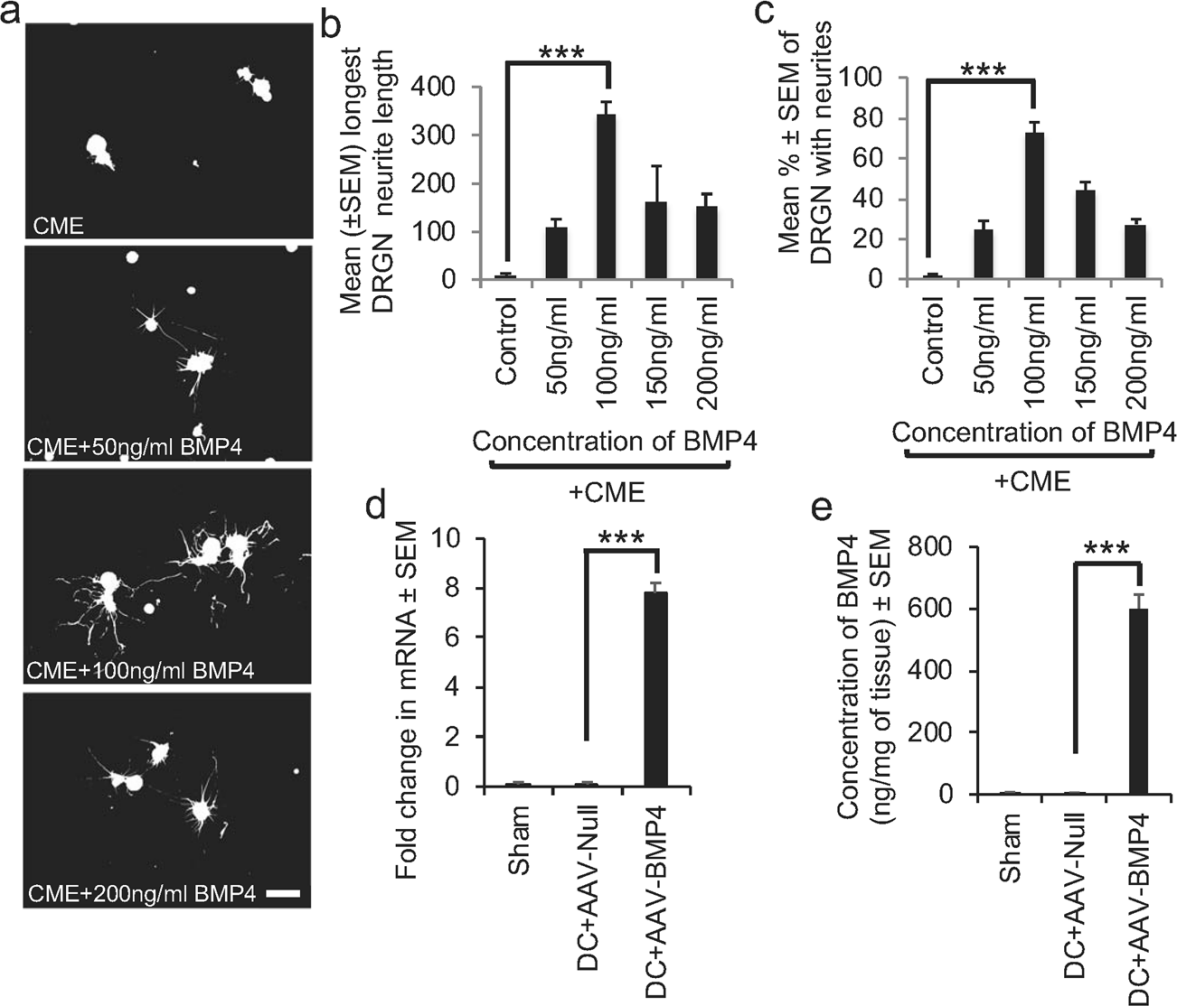
BMP4 disinhibits DRGN neurite outgrowth in vitro. **a** Escalating concentrations of BMP4 up to 100 ng/ml increased; **b** the length of the longest neurite; and **c** % DRGN with neurites. **d** Transduction with AAV-BMP4 significantly increased the levels of BMP4 mRNA by ~ 8-fold > DC + AAV-Null injection. **e** ELISA confirmed that AAV-BMP4 significantly increased the titres of BMP4 in DRG

There was no DRGN neurite outgrowth in control-untreated DRG cultures prepared from intact rats in the presence of inhibitory concentrations of CME (Fig. 3a), whilst positive control cultures treated with FGF2 showed significant disinhibited neurite outgrowth (not shown). However, increasing the concentration of BMP4 peptide, in the presence of CME, disinhibited DRGN neurite outgrowth, increasing mean DRGN neurite length (Fig. 3a, b) and the % DRGN with neurites (Fig. 3c) to a maximum at a concentration 100 ng/ml, beyond which disinhibited DRGN neurite outgrowth declined. These results showed that activation of the BMP4/Smad1 signalling pathway promoted DRGN survival and disinhibited neurite outgrowth in the presence of CME.

Intra-DRG injection of AAV-BMP4 significantly increased BMP4 mRNA levels by 7.6 ± 0.3-fold (Fig. 3d) whilst BMP4 protein levels also increased significantly to 642 ± 83 ng/mg of tissue, compared to 9.7 ± 1.2 ng/mg of tissue after DC + AAV-Null treatment (Fig. 3e). These results demonstrated that significant titres of BMP4 mRNA and protein were induced in DRGN after intra-DRG injection of AAV-BMP.

**Fig. 4:**
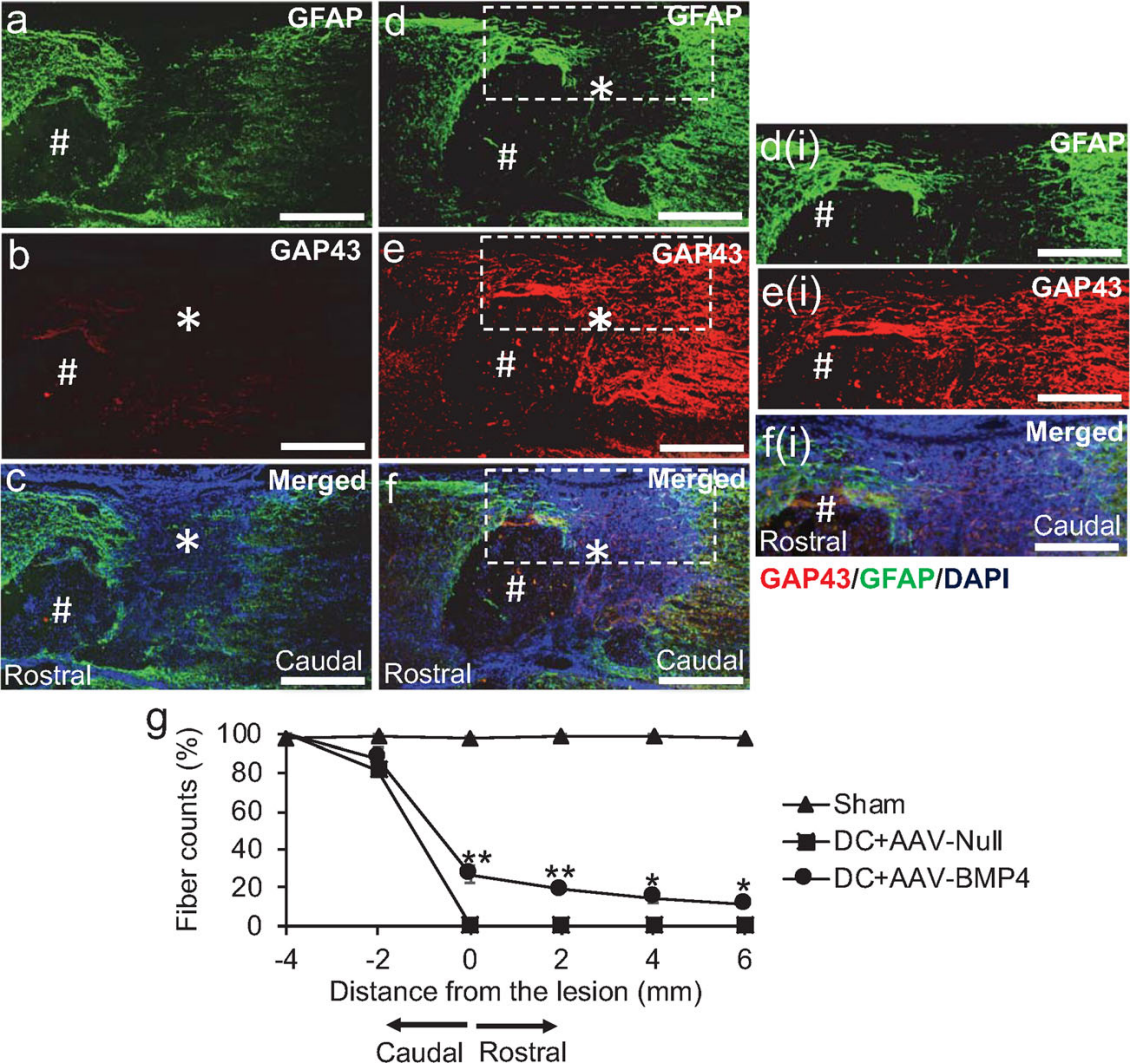
BMP4 promotes axon regeneration in an in vivo rat model that cavitates. **a**–**c** Treatment with DC + AAV-Null leads to cavity (#) formation at the lesion site (*) with no evidence of axon regeneration. **d**–**f** AAV-BMP4 enhances DC axon regeneration despite the presence of cavities (#), detected by GAP43^+^ staining (red) with GFAP^+^ astrocytes (green) also infiltrating the lesion site (*). **c**, **f** Merged images to show GFAP (green) and GAP43 (red) double staining with DAPI^+^ nuclei (blue). Inset **d(i)**, **e(i) and f(i)** = higher power views of the boxed regions in **d**–**f,** respectively. **g** Quantification of DC axon regeneration at the lesion site, rostral and caudal to the lesion. *** = *P* < 0.0001, ANOVA. ** = *P* < 0.001; * = *P* < 0.05, ANOVA. Scale bars in **a–f** and **d(i)**–**f(i)** = 100 μm

In DC + AAV-Null-treated rats (Fig. 4a–c), spinal cord cavitation (#) was observed at the lesion site (*) with GFAP^+^ immunoreactivity surrounding the lesion cavity (Fig. 4a), with little or no GAP-43^+^ (red) regenerating axons observed (Fig. 4b, c). However, intra-DRG injection of AAV-BMP4 (Fig. 4d–f) not only showed infiltration of GFAP^+^ astrocytes (green) into the lesion site (Fig. 4d, d(i) = high power of boxed region in Fig. 3d) but also promoted regeneration of ascending GAP43^+^ DC axons (red) into preserved tissue around areas of cavitation (#) about the lesion site (Fig. 4e, f; e(i), f(i) = high power of boxed regions in Fig. 4e, f, respectively). Quantification of the total number of GAP43^+^ axons (red) traversing the lesion site showed that 27 ± 4.4, 19.2 ± 2.2, 14.7 ± 2.4 and 11.33 ± 1.5% of axons regenerated 0, 2, 4 and 6 mm beyond the lesion site, respectively, in DC + AAV-BMP4-treated rats, whilst no axons were present in DC + AAV-Null-treated groups (Fig. 3g). These results suggested that AAV-BMP4 promoted DRGN axon regeneration in long-tract ascending pathways of the rat spinal cord, despite the presence of cavities.

### AAV-BMP4 Promoted Electrophysiological Recovery Across the DC Lesion Site

**Fig. 5:**
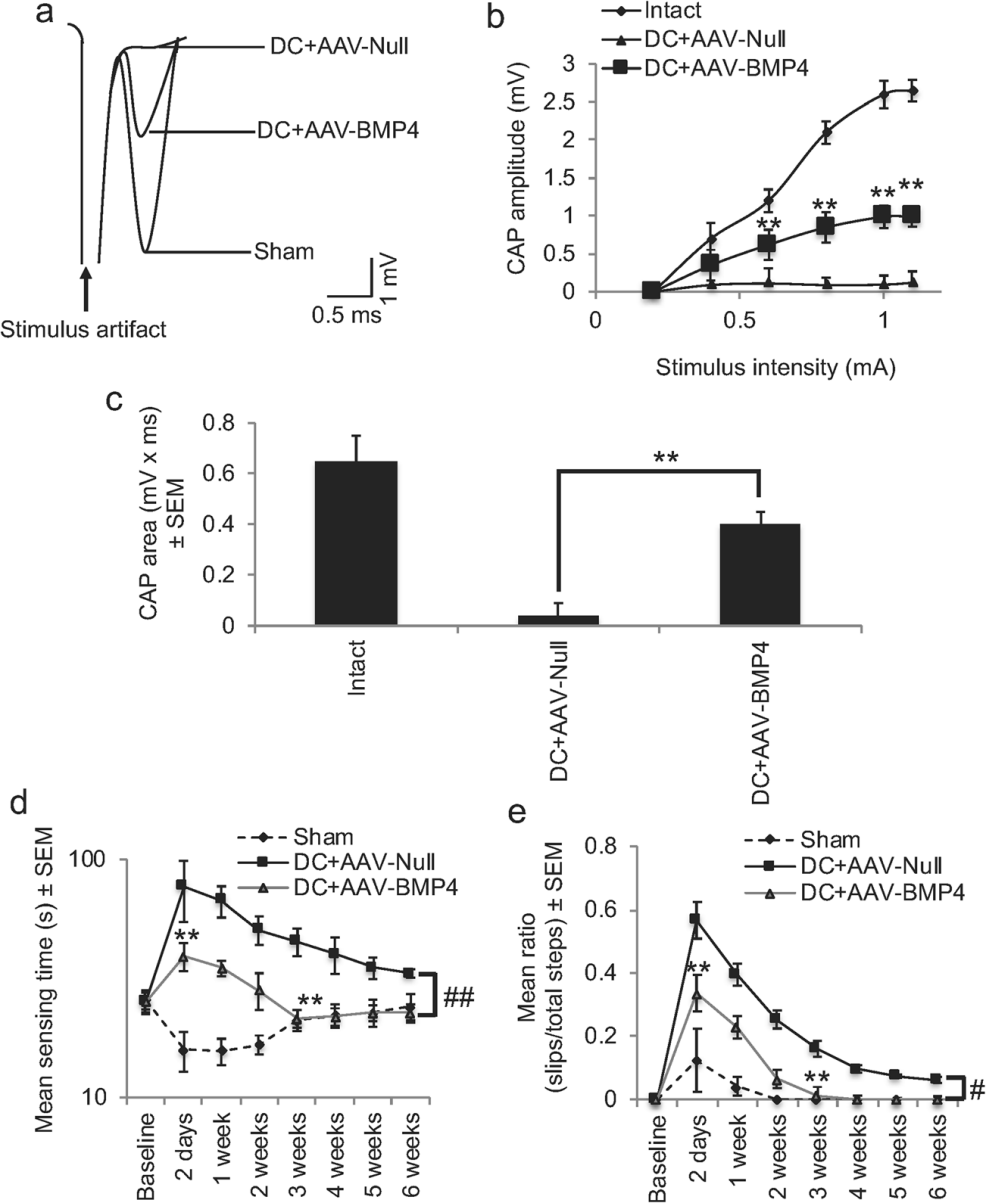
AAV-BMP4 promotes preservation of spinal CAP across the lesion site and functional recovery. **a** Superimposed CAP traces from representative animals in sham, DC + AAV-Null and DC + AAV-BMP4 treatment groups. **b** Compared to sham control, CAP amplitudes (mV) were greatly attenuated in the DC + AAV-Null group, but were significantly improved in the DC + AAV-BMP4 group (*P* < 0.001, ANOVA). **c** Mean CAP area at different stimulus intensities record from sham was significantly attenuated in the DC + AAV-Null group, but significantly improved in the DC + AAV-BMP4 compared to the DC + AAV-Null group (**P < 0.001, ANOVA). **d** Mean sensing time for the tape removal test and mean error ratios for the **e** horizontal ladder walking tests show significant recovery of function after AAV-BMP4 treatment. #*P* < 0.05; ##*P* < 0.001, generalised linear models. **P < 0.001, independent sample *t* test

Superimposed CAP traces from representative animals from the sham control, DC + AAV-Null and DC + AAV-BMP4 groups (Fig. 5a) showed that in the DC + AAV-Null groups, the mean amplitude of the CAP wave was significantly attenuated compared to that of the sham control group (Fig. 5b). The mean CAP amplitude was significantly reduced in the DC + AAV-Null group, compared to sham controls (Fig. 5b). However, significantly larger CAP amplitudes were observed in DC + AAV-BMP4-treated rats at all stimulation intensities, compared to those in the DC + AAV-Null treatment group (*P* < 0.001; Fig. 5b). In sham controls, CAP area (0.65 ± 0.1 mV × ms) was reduced to 8.0 ± 7.8% of the CAP area in DC + AAV-Null-treated groups (0.04 ± 0.05 mV × ms) (Fig. 5c). CAP area in the DC + AAV-BMP4 group was significantly larger (*P* < 0.001) than in the DC + AAV-Null group and was increased to 61.5 ± 10% of that of the sham control group (Fig. 5c). These results showed that in the AAV-BMP4 group, axons conduct action potentials across the lesion site and that conduction amplitudes had returned to > 60% of the original intact spinal amplitudes, despite the presence of cavities.

### AAV-BMP4 Promoted Functional Recovery

The mean sensing time for the tape removal test was between 12 and 25 s in sham-treated animals throughout the 6 weeks of testing (Fig. 5d). We observed a significant increase of 77 ± 22 s at 2 days in DC + AAV-Null-treated groups in the time it takes to sense and remove the adhesive tape decreasing to 39–52 s from 4 weeks onwards. However, in DC + AAV-BMP4-treated animals, the mean sensing time was significantly less at 2 days after injury when compared to DC + AAV-Null-treated animals, taking only 39 ± 6 s to detect and remove the adhesive tape (*P* < 0.001, independent sample *t* test). By 3 weeks after DC lesion, the mean sensing time in DC + AAV-BMP4-treated animals was no different to that of sham-treated control animals and significantly improved compared to DC + AAV-Null-treated animals (*P* < 0.001, independent sample *t* test). Over the whole-time course, there was a significant reduction in the time taken to sense and remove the adhesive tape in DC + AAV-BMP4-treated compared to DC + AAV-Null-treated animals (linear mixed model, *P* < 0.001).

Over a 6-week testing period, there was a significant increase in the error rates during horizontal ladder walking (generalised linear mixed model, *P* < 0.0011) (Fig. 5e) in DC + AAV-Null-treated compared to DC + AAV-BMP4-treated animals. The mean error ratio was significantly lower in DC + AAV-BMP4 compared to DC + AAV-Null-treated animals at 2 days after injury (*P* < 0.001, independent sample *t* test) and at 2–3 weeks (*P* < 0.001, independent sample *t* test) by which time the error rate was no different to that of sham controls. A significant functional deficit remained in DC + AAV-Null-treated rats throughout the duration of the test. These results demonstrated that AAV-BMP4 promoted significant sensory and locomotor function recovery after DC injury in the rat, despite the presence of cavities.

## Discussion

In this study, we investigated the relative contribution of signalling through the BMP4/Smad1 pathway in promoting long-tract DRGN axon regeneration after DC injury in the rat model that cavitates and showed by microarray and immunohistochemistry analyses that components of the BMP4/Smad1 pathway were highly correlative with the regenerative outcome seen after DC transection. For example, BMP4 and Smad1 mRNA and protein were highly upregulated in both regenerating paradigms whilst expression levels of the BMP4 suppressor Noggin were attenuated. Treatment of DRGN cultures with BMP4 significantly disinhibited neurite outgrowth in the presence of inhibitory concentrations of CME, whilst AAV8-mediated delivery of BMP4 in the rat in vivo promoted DC axon regeneration and electrophysiological, sensory and locomotor improvements after DC injury, despite the presence of cavities.

The PTEN/mTOR pathway has emerged as one key determinant in regenerative success in the adult RGC [29–31] and corticospinal tract [4, 32, 33]. However, *pten* deletion not only promotes axon regeneration through mTOR-dependent mechanisms [30] but also through mTOR-independent mechanisms [34]. Numerous other studies have shown that mTOR activity is not required for peripheral nerve regeneration [35–37]. Instead, peripheral sensory axon regeneration is thought to be mediated by PI3K signalling via GSK3 inactivation and subsequent gene expression, independent of mTOR-mediated protein synthesis [37, 38].

In DRGN, a possible PI3K/Akt axogenic signalling route, independent of mTOR, is through the BMP4/Smad1 pathway which influences a broad spectrum of intracellular signalling pathways [12, 39, 40] including MAPK, GSK3-β, PI3K and Akt. The DRGN axon growth promoted by NTF/Trk binding and subsequent activation of the MAPK and PI3K/Akt effector pathways is arrested after suppression of BMP [13] probably because, in the absence of Smad1, transcription of the NTF effectors Erk1/2 is blocked [10]. Within the BMP/Smad pathway, raised BMP4 and Smad1, 2, 4, 5, 8 mRNA and lowered Noggin mRNA levels correlated with complimentary changes in BMP4, pSmad1 and Noggin protein in DRGN in the DC, SN and pSN + DC models. Moreover, our observation that BMP4 disinhibited DRGN neurite growth on a CME substrate in culture agrees with previous findings [15]. Both SN axotomy and intra-DRG injection of BMP2/4 protein activates Smad1 in DRGN and enhances DRGN neurite outgrowth, whilst DC transection fails to activate the Smad1 pathway [14].

Down-regulation of the BMP/Smad1 pathway occurs during the age-related decline in axon growth potential and, in adults, blockade of BMP signalling, by either pharmacological inhibition or knockdown of Smad1, arrests the initiation and elongation of DRGN neurites [13], whereas activation of Smad1 by intrathecal injection of AAV-BMP4 stimulates DRGN axon regeneration through mouse DC lesions [13]. However, BMP control of growth cone mobility through regulation of actin dynamics is independent of Smad1 and involves direct interaction between the tail region of BMPRII and LIMK [41–44]. Levels of Noggin mRNA were reduced in the SN and pSN + DC axon regenerating paradigms but not in the non-regenerating DC, agreeing with findings that Noggin inhibits BMP signalling by blocking type I/II BMPR binding sites [45] and reducing DRGN neurite outgrowth. Conversely, suppression of Noggin potentiates SN axon regeneration in vivo [15].

Despite contradictory reports that BMP4 inhibits axon regeneration by promoting hypertrophic scarring [46] and that Noggin promotes axon growth [15, 47] in the spinal cord, we and others [13] have demonstrated DC axon regeneration and functional recovery after delivery to DRGN of either BMP4 peptide or AAV8*-*BMP4 after intra-DRG and intrathecal injection in both mouse and rat. Moreover, there was constitutive expression of Noggin in DRGN in the DC, but a 2-fold reduction in the SN and pSN + DC regenerating models, indicating that future delivery to DRGN of a combination of AAV8-BMP4 with a Noggin antagonist is expected to significantly enhance DC axon regeneration further and promote significant functional recovery, as shown by the preservation of CAP and improvements in ladder walking and tape removal tests. Our electrophysiological data recorded cord dorsum potentials, which not only measured dorsal column activity but may have detected signals from diverse sources. For example, CDP is an evoked spinal cord field potential that arises in the dorsal horn interneurons of the spinal cord segments receiving inputs from a stimulated peripheral nerve and can originate from proximal sensory nerve, dorsal nerve root and spinal cord dorsal horn function [48]. Therefore, it is likely that some improvements may be due to preservation of these pathways in the AAV-BMP4-treated groups.

The beneficial effects of BMP4 delivery demonstrated in our study are significant since previous work by Parikh et al. (2011) using the same AAV8-BMP4 promoted DC axon regeneration and functional recovery in the mouse, which does not cavitate but instead fills the lesion site with fibrotic tissue [16, 49]. SCI in the rat normally results in large cystic cavities that extend rostrally and caudally to the original lesion site [50]. Pathologically, rat SCI is more similar to that seen in humans where spinal cord atrophy, myelomalacia, cyst and syrinx formation occurs [51]. Therefore, our demonstration of the benefits of BMP4 treatment after SCI in the rat is translationally more relevant to the human condition.

Although we used AAV8 to deliver BMP4, which is not considered translational since transgenes were injected 1 week before DC injury, the study does demonstrate proof-of-principle that activation of the BMP4/Smad1 pathway is important in promoting long-tract ascending DC axon regeneration after injury. However, we have shown in the rat DC lesion model that a non-viral delivery vector, in vivo-JetPEI, delivered plasmid DNA with the same efficiency of transduction as AAV and without activation of non-specific interferon responses, promoting similar DC axon regeneration, electrophysiological and functional recovery [19]. We expect that the same non-viral delivery vector can be used to safely deliver BMP4 and activate the BMP/Smad1 signalling pathway, making the approach translationally relevant.

Thus, we have provided in vitro and in vivo evidence that the BMP4/Smad1 pathway was activated in DRGN in the regenerating SN and pSN + DC models but not in the non-regenerating DC model. In vitro, BMP4 peptide disinhibited significant DRGN neurite outgrowth and, in vivo, AAV8-BMP4 promoted DC axon regeneration and functional recovery without the need for an SN pre-conditioning lesion, in a rat model of SCI that cavitates like in humans. We conclude that BMP4 over-expression promoted significant DRGN axon regeneration and enhanced recovery of lost function in the lesioned DC and may be a potential therapy for SCI patients.
